# HS-GC-IMS Analysis of Volatile Organic Compounds in Six Spicy Spices and Their Effects on Ulcerative Colitis

**DOI:** 10.3390/molecules29163764

**Published:** 2024-08-08

**Authors:** Qi Gao, Qiang Zhang, Chunliang Wang, Xue Geng, Min Hua, Nianhong Li, Yanpeng Dai, Yan Zhang, Qian Zhou

**Affiliations:** 1 Shandong Academy of Chinese Medicine, Jinan 250014, China; gq20211122@163.com (Q.G.); zq809128349@163.com (Q.Z.); huamlkh@163.com (M.H.); nianhongli2022@163.com (N.L.); daiyanpeng1027@163.com (Y.D.); zh01984@126.com (Y.Z.); 2 Shandong Modern Research and Development Engineering Center of Traditional Chinese Medicine Aromatherap, Jinan 250014, China; 3 Qilu Pharmaceutical Co., Ltd., Jinan 250100, China; chunliang.wang@qilu-pharma.com; 4 School of Pharmacy, Shandong University of Traditional Chinese Medicine, Jinan 250355, China; gengxue5927111@163.com; 5 NMPA Key Laboratory for Research and Evaluation of Generic Drugs, Shandong Institute for Food and Drug Control, Jinan 250101, China

**Keywords:** spices, HS-GC-IMS, volatile organic compounds, ulcerative colitis, zebrafish

## Abstract

The volatile organic compounds of six spices, including black pepper, dried ginger, cinnamon, fennel, clove, and zanthoxylum, were analyzed by gas chromatography–ion mobility spectrometry (HS-GC-IMS) combined with principal component analysis (PCA) and Euclidean distance. In further analyses, the effects of volatile oils in six spices on ulcerative colitis were assayed in a zebrafish model induced by 3-nitrobenzenesulfonic acid. A total of 120 kinds of volatile organic compounds were detected and 80 among them were identified, which included 10 common components and 3 to 24 characteristic components belonging to different spices. The major VOCs in six spices were estimated to be terpenes with the contents of 45.02%, 56.87%, 36.68%, 58.19%, 68.68%, and 30.62%, respectively. Meanwhile, the volatile components of fennel, dried ginger, black pepper, and cinnamon are quite similar, but differ from clove and zanthoxylum. The volatile oils in six spices presented efficient activity to improve ulcerative colitis which can decrease the number of neutrophils, restore the structure of intestinal epithelial and the morphology of the epithelial cells. Our study achieved rapid analysis of the volatile organic compounds and flavors in six spices and further revealed the potential health benefits of their volatile oils on ulcerative colitis, especially for clove and zanthoxylum. This study is expected to provide certain data support for the quality evaluation and the potential use in functional foods of six spices.

## 1. Introduction

Spices have been widely used worldwide for thousands of years, which can add flavor to foods due to their unique colors and aroma, and they are one of the important ingredients or seasoning in cooking and the food industry. For example, cinnamon is usually used in desserts bakery and in the preparation of beverages such as cola and wine in the European diet culture [1]. In China, spices also show common application. They play a cardinal role in the marinating of meat, processing of traditional Chinese food such as Dezhou braised chicken and hotpot, as well as in stir-fries, boiling, and cold dressing of daily cooking [2,3]. In addition to being used as condiments, most spices are edible and medical resources. They have certain special medicinal values and have a good effect on daily health care. So far, many bioactivities including anti-inflammatory [4], antitumor [5], antioxidant [6], and antibacterial [7] have been reported. It was also pointed out that spices have protective effects on the cardiovascular system, digestive system, and central nervous system [5,8,9,10]. Black pepper, dried ginger, cinnamon, fennel, clove, and zanthoxylum are the most commonly used spices in the Chinese diet, which can give foods special colors and flavors, promote intestinal peristalsis, and increase appetite [11,12]. According to the theory of traditional Chinese medicine, these six spices are interior-warming medicines and widely used in the treatment of digestive system diseases due to their warm nature [13,14,15,16,17,18]. What is more, Kondapalli, N.B. et al. [19] found that the extracts of black pepper can regulate gut microbiota modulations and decrease the IL-6 level in serum to achieve the effects of anti-inflammatory. Min Fu et al. [14] investigated the coptis chinensis and dried ginger herb combination, which can inhibit gastric tumor growth by interfering with glucose metabolism via LDHA and SLC2A1. Shuguang Yan et al. [20] found that cinnamaldehyde contained in cinnamon can alleviate aspirin-induced gastric mucosal injury by regulating pi3k/akt pathway-mediated apoptosis, autophagy, and ferroptosis. The spicy odor due to the volatile organic compounds contained in these spices not only determine the sensory characteristics of spices but also connect tightly with the properties of anti-inflammatory, antioxidant, and anti-diabetic [21,22,23]. Volatile compounds are extremely important for better development and utilization of warm spices in the food industry and the TCM clinical application, which has undoubtedly aroused great interest in the study of them.

Currently, GC-MS, GC-IMS, GC-O-MS, and E-nose techniques are commonly used for detecting volatile organic compounds [24]. GC-MS is required to improve the sensitivity of instrument, but its operation is complicated. E-nose can achieve rapid analysis of volatile organic compounds, but the lower reproducibility of the results limits its practical application [25]. Headspace gas chromatography–ion mobility spectrometry (HS-GC-IMS) combines the high separation capacity of gas chromatography and the rapid response of ion mobility spectrometry, which has the advantages of separating volatile organic compounds with high efficiency, rapid response, and high sensitivity. HS-GC-IMS is easy to operate as its samples do not require pretreatment, and the results can reflect the differences among samples intuitively [26]. In recent years, HS-GC-IMS has been widely used for the study of volatile organic compounds in foodstuffs, Chinese medicine, medical testing, and environment-protective characteristics [27], such as identification of Ophiopogonis Radix from different producing areas [28], the flavor substance changes in green tea during storage [29], and the influence of cooking methods on volatile flavor compounds in green onion [30]. Compared to GC-MS, HS-GC-IMS has become the first choice for fast chemical profiling of volatile compounds in solid matrix and quality control purposes. And it has the advantage of analyzing volatile components among multiple samples in parallel. Most importantly, HS-GC-IMS can combine the high separation capacity of GC with high selectivity of IMS for the analysis of volatile organic compounds, as with the ability of efficient separation according to ion mobility at atmospheric pressure, which is particularly beneficial to identity the isomers’ volatile compounds.

Ulcerative colitis (UC) is a diffuse inflammatory disease of the rectal and colonic mucosa with the characteristic of chronic or recurrent, which leads to the injury of tissue with bowel inflammation and ulceration [31,32]. At present, the treatment of ulcerative colitis has the disadvantages of high cost and side effects, while some natural active products show excellent anti-inflammatory activity [33,34] or other function with high efficiency and low toxicity. So, it is of great significance to explore active substances effective for ulcerative colitis from natural resources. The previous studies have also confirmed that some spices have gastrointestinal-protective activities, such as anti-gastric ulcer and aiding in digestion [2,35].

Zebrafish (Danio rerio) is a powerful model system in biomedical research due to their genetic tractability, external development, and optical transparency during the embryonic and early larval stages [36]. Moreover, zebrafish have been widely used in research works of intestinal physiology and disease because the zebrafish intestine closely resembles the mammalian intestine [37]. When exposed to 2,4,6-trinitrobenzen sulfonic acid (TNBS), an agent commonly used to induce ulcerative colitis, the phenotypes exhibited by zebrafish are similar compared to mammals, including severe destruction of the epithelium and a strong inflammatory response [38]. Hence, we choose the zebrafish model to assess the effects of volatile oils in six spices on TNBS-induced intestinal inflammation.

In recent years, black pepper, dried ginger, cinnamon, fennel, clove, and zanthoxylum have gained much attention and popularity due to their better edible and medicinal values. However, there is a lack of systematic analytical research on the volatile components contained in warm spices. Indeed, the differences in flavor among different spices are not fully understood. Moreover, most studies on spices focus on the effects of spices in powder form and there are few studies that systematically evaluate the effects of volatile components contained in a certain type of spice on ulcerative colitis. Thus, we choose the six warm spices mentioned above to analyze the similarities and differences of the volatile components contained in them and explore their effects on ulcerative colitis based on the zebrafish model. This study will provide data support for the quality evaluation and the development of functional food products about warm and spicy spices.

## 2. Results and Discussion

### 2.1. Spectral Analysis of Six Spices

**Figure 1:**
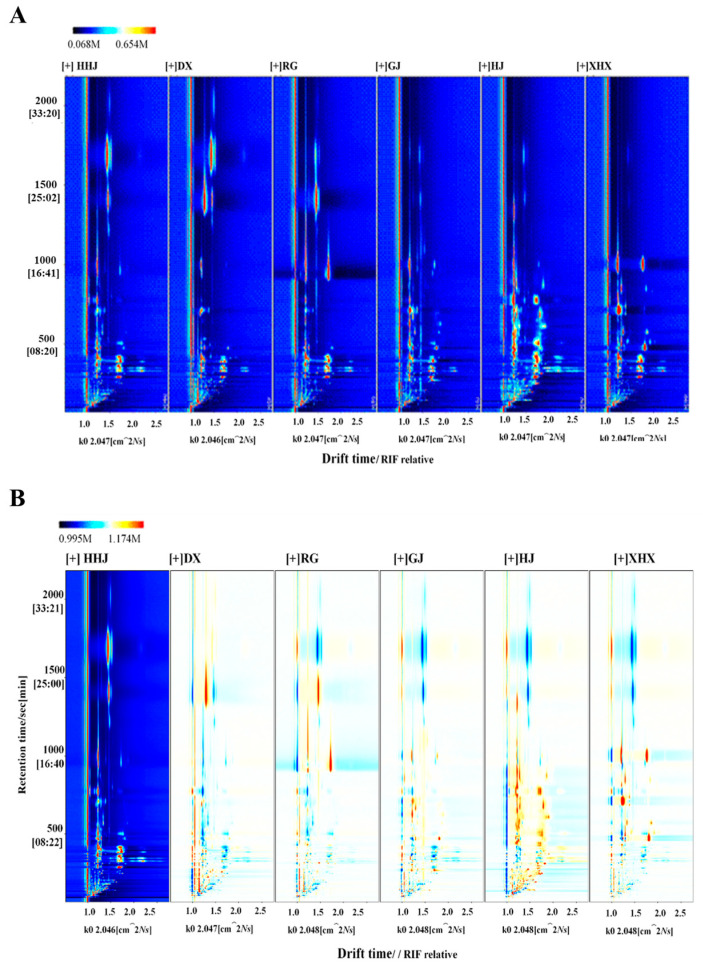
Topographic plots of six spices (**A**) and difference diagram of six spices using HHJ as reference deduction (**B**).

A two-dimensional top view of the VOCs in HHJ, HJ, DX, RG, XHX, and GJ was plotted to comprise the differences in VOCs between the different spices (Figure 1). The X- and Y-axes represent the ion migration time and retention time of GC, respectively, which could be used for qualitative analysis of each component. The red vertical line at abscissa 1.0 was the peak of reaction ion (RIP). The different colored dots to the right of the RIP represent the different VOCs detected. The concentration of VOCs was represented by different colors of dots in the plot. The dots with white color meant lower concentration of VOCs, while the red one meant higher concentration of volatiles. And the darker the color indicated, the greater concentration of detected VOCs. As shown in Figure 1A, most of the signals occurred at the retention time of 100–1000 s, drift time of 1.0–2.0 ms, and more VOCs were detected in HJ than in other samples.

In order to further compare the differences of six spices, HHJ was selected as the reference contrast and the remaining samples minus the reference. If the background after subtraction was white, it meant that the two VOCs agree. If it was red, it indicated that the concentration of the substance was higher than the reference, while blue meant it was lower than the reference (Figure 1B). The similarities and differences of VOCs in six spices can be compared clearly in this contrast model. In the range of retention times of 50–500 s and drift times of 1.0–1.2 ms, the differences of VOCs in DX, RG, GJ, XHX, and HHJ were smaller, while there were significant differences between HJ and HHJ. Under the same conditions, there were more VOCs detected in HJ than other samples, which is consist with its obvious aroma.

### 2.2. Identification of Volatile Organic Compounds in Six Spices

There were 120 signal peaks detected in six spices by comparing their IMS drift times and retention indices with authentic reference compounds. A total of 80 compounds were identified from the topography by the GC-IMS library, including 16 terpenes, 14 alcohols, 12 ketones, 23 aldehydes, 1 acid, 3 ethers, 9 esters, and 2 aromatic compounds. Of these, citral, ethyl phenylacetate, heptaldehyde, 2-heptanone, trans-2-hexenal, hexanal, pentanal, 2-methylbutyraldehyde, 3-methylbutyraldehyde, ethyl acetate, 2-methyl propanol, isobutyraldehyde, 2-propenylmethy disulfide, isoamyl acetate, furfural, 2-butanone, 2-pentanone, butyl sulfide, 2-nonanone, and benzaldehyde were present as monomers and dimers, which may produce multiple signals or spots in HS-GC-IMS profiles due to their varying concentrations [39,40]. M and D represented monomer and dimer in Table 1, respectively.

Thirteen compounds were identified in HHJ, where terpenes (45.02%) have the highest concentration. Sixteen compounds were identified in RG, in which terpenes (17.45%) and alcohols (36.68%) showed a higher content. Thirty-four compounds were identified in HJ, and of these, terpenes (30.62%) and alcohols (29.58%) were higher than other components. Twenty-two compounds were identified in DX, and most of them were terpenes (68.68%). In GJ, 24 compounds were identified which consisted largely of terpenes (56.87%) and aldehydes (19.1%). And 21 compounds were identified in XHX, which mainly consisted of terpenes (58.19%) and aldehydes (29.69%). The volatile organic components in six spices were mainly comprised of terpenes, which were closely related to the odor characteristics of spices. And the content and types of terpenes were also different among the six spices. Therefore, we speculated that terpenes can be considered as biomarkers for quality evaluation and flavor analysis of spices.

Most studies focused on analyzing volatile components in volatile oils that were extracted from spices through GC-MS. However, little attention has been paid to analyze the spices directly. Compared with the analysis results about corresponding volatile oils reported in the literature, the HS-GC-IMS results of HHJ, RG, HJ, DX, GJ, and XHX in this study showed some similarities and differences [41,42,43,44,45,46]. Dosoky, N.S. et al. [42] analyzed volatile oils of black pepper by GC-MS, and the results showed that the major components of the volatile oils were terpenes, such as α-pinene, δ-2-carene, and limonene. In this study, terpenes were also the major volatile components in HHJ, but the characteristic volatile compounds detected were different. For example, the GC-MS results showed that the components with high content in black pepper oils were α-pinene, δ-2-carene, limonene, sabinene, and phellandrene, which were also detected in this study. However, the components, such as β-caryophyllene, β-pinene, and linalool, were not detected. Najdoska-Bogdanov et al. adopted SHS-GC-FID to analyze the volatile components in fennel. Comparing two studies, both estragole and anethole were detected in fennel, while *p*-Mentha-1(7),8-diene, sabinene, phellandrene, trans-β-ocimene, and nerol were also detected in this study. The differences mentioned above may be related to the limitation of the IMS database, the efficiency of the chromatography column, and the source of samples. It can be seen that GC-MS, GC-FID, HS-GC-IMS, and other methods can be used complementally to achieve more comprehensive analysis of volatile components. However, HS-GC-IMS is a better choice when rapid analysis is required in practice.

### 2.3. Fingerprints of VOCs in Six Spices

**Figure 2:**
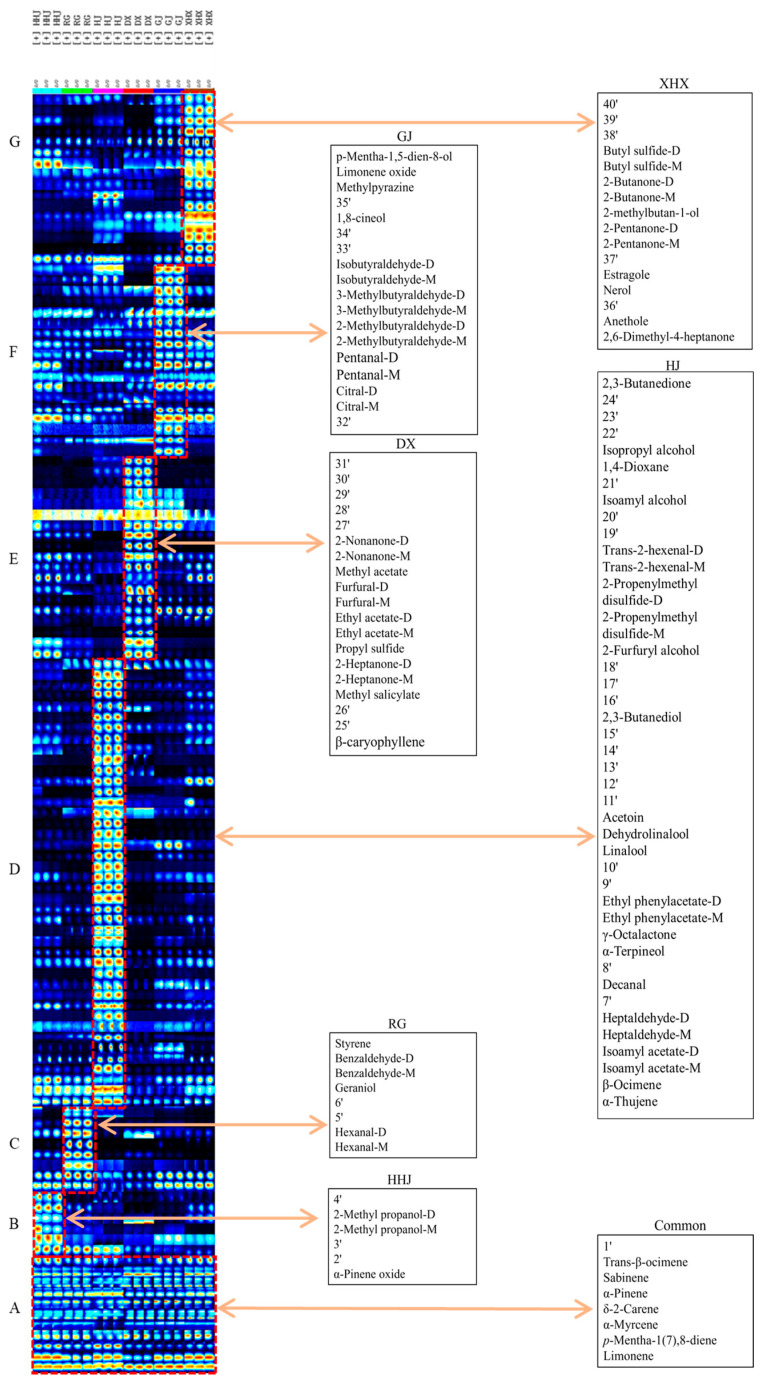
Fingerprints of six spices generated by Gallery Plot. (**A**) the commmon VOCs in six spices. (**B**): the characteristic volatile components of HHJ; (**C**): the characteristic volatile components of RG; (**D**): the characteristic volatile components of HJ; (**E**): the characteristic volatile components of DX; (**F**): the characteristic volatile components of GJ; (**G**): the characteristic volatile components of XHX. The unknown compounds are presented by **1′**–**40′**, respectively.

In order to compare the similarities and differences of characteristic VOCs in six spices, the fingerprints were generated by Gallery Plot plug-in of the software (Version number: 2.2.1) (Figure 2). All signals that could be detected under experimental conditions are shown in Figure 2, in which each row represents the substance detected in one sample and each column represents the signal intensity of the same volatile compounds in different samples. Each dot in Figure 2 represents a kind of volatile organic compound, which contents were related to the color of the dots. The brighter the color, the higher the level, and vice versa. The compound number in each area is consistent with that in Table 1, and **1′**–**40′** represent unknown components, as shown in Figure 2.

Box A (**42**, **43**, **16**, **15**, **17**, **18**, **21**, **19**, **22**, **68**, **1′**) contained the common VOCs in six spices which were limonene, α-phellandrene, *p*-Mentha-1(7),8-diene, α-myrcene, α-pinene, δ-2-carene, sabinene, acetone, ethanol, trans-β-ocimene, respectively. And there were no significant differences in the content of them among the samples.

Box B (**14**, **2′**, **3′**, **39**, **52**, **4′**) contained six characteristic volatile components of HHJ, of which three could be identified, including α-pinene oxide, 2-methyl propanol, and 2-methyl propanol dimer. And limonene, α-phellandrene, and α-myrcene were the major aroma compounds that were contained in the volatile components of HHJ.

The characteristic volatile compounds of RG were contained in Box C (**29**, **30**, **5′**, **6′**, **6**, **60**, **67**, **69**), including hexanal (D), hexanal (M), geraniol, benzaldehyde (M), benzaldehyde (D), and styrene. Terpenes play an important role in the aroma of cinnamon. For example, sabinene is one of the major aroma components which has the flavor of wood and pungent.

As shown in Box D (**23**, **20**, **46**, **47**, **24**, **25**, **7′**, **4**, **8′**, **7**, **12**, **5**, **13**, **9′**, **10′**, **65**, **64**, **50**, **11′**, **12′**, **13′**, **14′**, **15′**, **70**, **16′**, **17′**, **18′**, **73**, **44**, **72**, **28**, **74**, **19′**, **20′**, **75**, **21′**, **76**, **78**, **22′**, **23′**, **24′**, **77**), a total of forty-two characteristic volatile compounds were detected in HJ, of which twenty-four were identified, including α-thujene, β-ocimene, isoamyl acetate (M), isoamyl acetate (D), heptaldehyde (M), heptaldehyde (D), decanal, α-terpineol, γ-octalactone, ethyl phenylacetate (M), ethyl phenylacetate (D), linalool, dehydrolinalool, acetoin, 2,3-butanediol, furfuryl alcohol, 2-propenylmethyl disulfide (M), 2-propenylmethyl disulfide (D), trans-2-hexenal (M), trans-2-hexenal (D), isoamyl alcohol, 1,4-dioxane, isopropyl alcohol, and 2,3-butanedione, arranged from left to right. Zanthoxylum has the aroma characteristics of flower, sweet, pepper, wood, and green, which is mainly related to the alcohols and terpenes, such as linalool and limonene.

The characteristic compounds of DX are shown in Box E (**1**, **25′**, **26′**, **8**, **26**, **27**, **45**, **37**, **38**, **48**, **49**, **53**, **62**, **59**, **27′**, **28′**, **29′**, **30′**, **31′**), including β-caryophyllene, methyl salicylate, 2-heptanone (M), 2-heptanone (D), propyl sulfide, ethyl acetate (M), ethyl acetate (D), furfural (M), furfural (D), methyl acetate, 2-nonanone (M), and 2-nonanone (D). β-caryophyllene has the flavor of wood and pungent, which is one of the essential factors affecting the aroma characteristics of clove.

The compounds contained in Box F (**32′**, **2**, **3**, **32**, **51**, **33**, **34**, **35**, **36**, **40**, **41**, **33′**, **34′**, **66**, **35′**, **71**, **79**, **80**) were the characteristic components detected in GJ, including citral (M), citral (D), pentanal (M), pentanal (D), 2-methylbutyraldehyde (M), 2-methylbutyraldehyde (D), 3-methylbutyraldehyde (M), 3-methylbutyraldehyde (D), isobutyraldehyde (M), isobutyraldehyde (D), 1,8-cineol, methylpyrazine, limonene oxide, and *p*-mentha-1,5-dien-8-ol. The terpenes, such as 1,8-cineol, α-phellandrene, α-pinene, and sabinene, have the odor of mint, sweet, and turpentine, which is closely related to the spicy, sweet, mint, fruity, and grassy aromas of ginger [44].

The characteristic compounds of XHX are shown in Box G (**61**, **11**, **36′**, **9**, **10**, **37′**, **56**, **57**, **31**, **54**, **55**, **63**, **58**, **38′**, **39′**, **40′**), including 2,6-dimethyl-4-heptanone, anethole, nerol, estragole, 2-pentanone (M), 2-pentanone (D), 2-methylbutan-1-ol, 2-butanone (M), 2-butanone (D), butyl sulfide (M), and butyl sulfide (D). Of these, anethole is the characteristic aroma component which has the smell of sweet and anise [47]. Trans-β-ocimene has the flavor of sweet and herb. α-phellandrene, α-pinene, sabinene, and butyl sulfide have the flavor of spice, pine, turpentine, or herb. The components mentioned above give fennel a characteristic aroma of strong and sweet.

The above results indicated that the similarities and differences of six spices can be shown by HS-GC-IMS, clearly. And the detected volatile organic compounds are closely related to the flavor of spices. In addition, we can find that more of the isomers, e.g., alpha and betta pinene, were detected by HS-GC-IMS than by GC-IMS. This is mainly attributed to the efficient separation according to ion mobility accomplished at atmospheric pressure [48], which means that HS-GC-IMS can serve as an effective approach for the discrimination of isomers’ volatile compounds.

### 2.4. Cluster Analysis of VOCs in Six Spices

#### 2.4.1. Dynamic PCA of Six Spices

Principal component analysis (PCA) is a method based on multivariate statistics to reduce the dimension of data or transform multiple indicators into a few comprehensive indicators for obtaining characters and revealing the relationship between variables [49]. In this study, PCA analysis was performed on VOCs in six spices by MATLAB R2018a. Eigenvalue and variance contribution rate were the indicators to choose principal components. As shown in Table 2, the initial eigenvalues of the five principal components were all above 1, and the variance contribution rates of them were 34.241%, 23.228%, 15.954%, 10.383%, and 8.470%, respectively. The cumulative variance contribution rate of five principal components was 100%, which could reflect the whole information of the samples and can be chosen as the effective components for data analysis [50].

The relative content of volatile organic compounds was standardized by MATLAB R2018a to obtain principal component scores and comprehensive scores (Table 3). As shown in Table 3, There were significant differences in comprehensive scores of principal components, of which HJ had the highest scores, followed by GJ, HHJ, RG, DX, and XHX.

**Figure 3:**
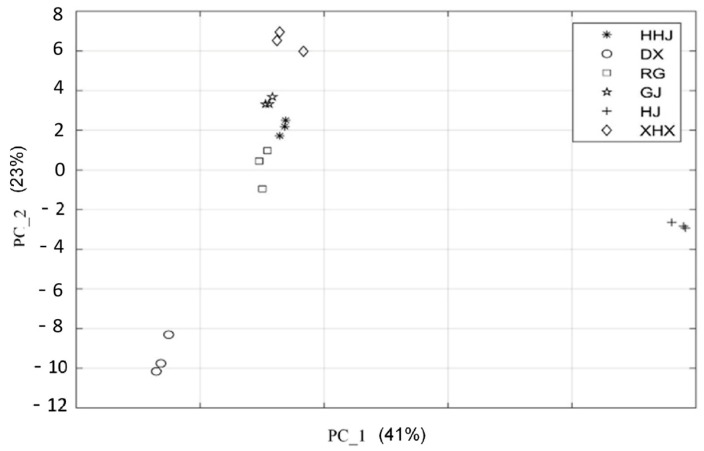
PCA analysis on volatile compounds of six spices.

The principal component analysis results of six spices are shown in Figure 3, in which the dots with different colors represent different samples of spices, and the distance between individual dots represents the level of similarity. In Figure 3, XHX, GJ, HHJ, and RG are grouped together, while HJ and DX are scattered in area with a far distance. This indicated that the VOCs of XHX, GJ, HHJ, and RG were similar, which were significantly different from those of HJ and DX. Meanwhile, HJ and DX dispersed in different quadrants, which meant that there were differences in their VOCs. According to Figure 3, PC 1 accounts for 41% and PC 2 accounts for 23%, with a total cumulative contribution of 64% by the two principal components, which indicated that the data of HS-GC-IMS involve effective information that could distinguish the six different spices.

#### 2.4.2. Analysis Based on Euclidean Distance

**Figure 4:**
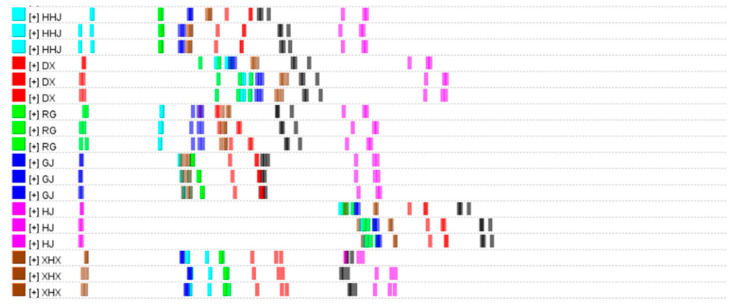
Euclidean distance of six spices.

Euclidean distance is a method of cluster analysis. It is possible to evaluate the quality of two samples by applying the Euclidean distance similarity algorithm. And it has been verified that the algorithm can accurately and reliably evaluate the quality of samples [51]. The similarities are determined by the distance coefficient. The larger the distance coefficient between samples, the larger the difference between them. On the contrary, the smaller the distance coefficient, the smaller the differences of the two, and the greater the similarity. The Euclidean distance results of six spices are shown in Figure 4. XHX, GJ, RG, and HHJ are close to each other but far from DX and HJ, which indicated that XHX, GJ, RG, and HHJ had higher similarity than DX and HJ. The results were consistent with PCA.

### 2.5. Improvement of Volatile Oils from Six Spices on Ulcerative Colitis in Zebrafish

**Figure 5:**
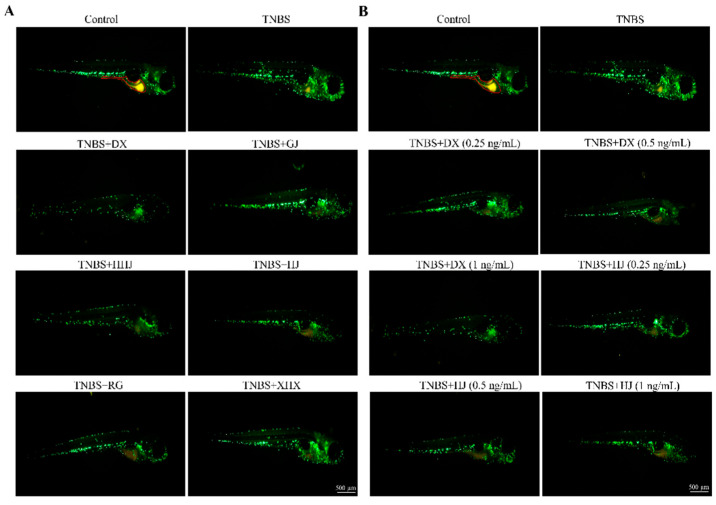

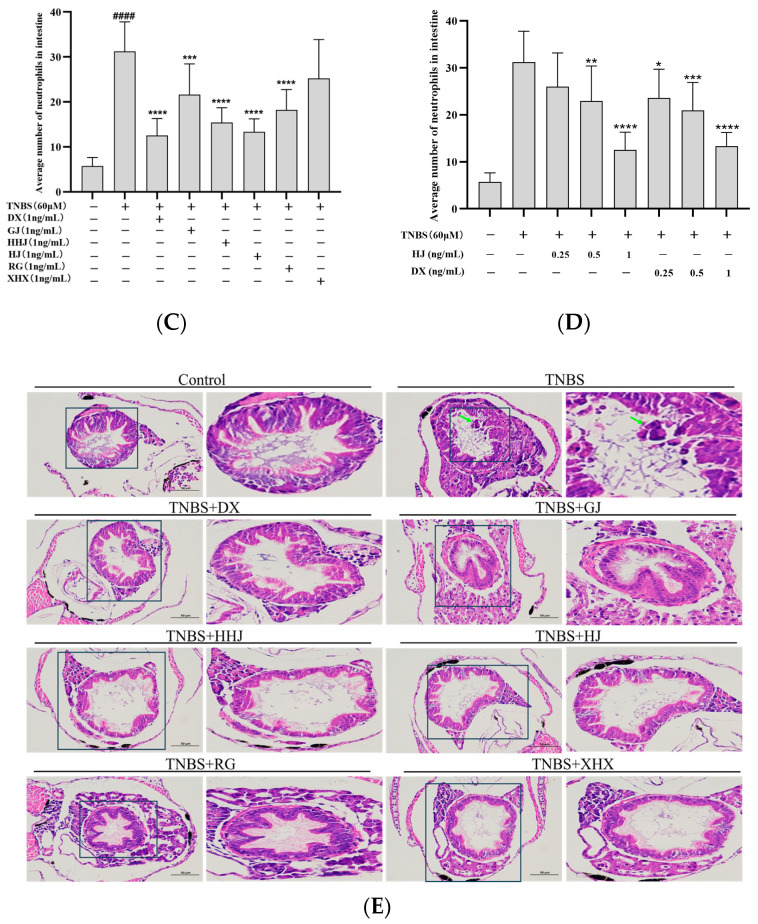
The effects on ulcerative colitis in zebrafish of volatile oils. (**A**) The diagram of intestinal fluorescence phenotypes in each group (*n* = 10, the concentration of TNBS was 60 μM and the concentration of volatile oils was 1 ng/mL, respectively). Scale bar is 500 μm. (**B**) The average number of neutrophils in zebrafish in each group. Significant difference was compared with the control or TNBS group, *** *p* < 0.001, **** *p* < 0.0001 vs. model group, #### *p* < 0.0001 vs. control group (*n* = 10). (**C**) The diagram of intestinal fluorescence phenotypes in the groups treated with DX or HJ volatile oils of different concentrations (*n* = 10, the concentration of TNBS was 60 μM). Scale bar is 500 μm. (**D**) The average number of neutrophils in zebrafish in the groups treated by DX or HJ volatile oils with different concentrations. Significant difference was compared with the control or TNBS group, * *p* < 0.05, ** *p* < 0.01, *** *p* < 0.001, **** *p* < 0.0001 vs. model group (*n* = 10). (**E**) Effect of six volatile oil samples on the histopathological structure (HE staining (green arrow)) of intestinal in ulcerative colitis zebrafish (*n* = 10, the concentration of TNBS was 60 μM and the concentration of volatile oils was 1 ng/mL, scale bar is 50 μm).

Neutrophils are an essential part of ulcerative colitis (UC) pathogenesis. Neutrophilic infiltration is a hallmark of UC histopathology and the potential pharmacological target for therapeutic intervention [52]. The effects of volatile oils extracted from six spices on neutrophils in intestine of zebrafish are shown in Figure 5A–D. Compared with the control group, the neutrophils in zebrafish intestine significantly increased (*p* < 0.0001) in the model group treated by TNBS, which indicated that the models were established successfully. After being treated by volatile oils of six spices with the concentration of 1 ng/mL, compared with the model group, the average number of neutrophils in the black pepper, zanthoxylum, fennel, cinnamon, clove, and dried ginger groups decreased by 50.6%, 57.4%, 19.2%, 41.7%, 59.9%, and 30.8%, respectively. The number of neutrophils significantly decreased (*p* < 0.0001, *p* < 0.001 vs. model group), except for the fennel group (Table 4). The results showed that the volatile oils of six spices have significant activity in improving ulcerative colitis of zebrafish, in which the groups treated by DX and HJ oils exhibited the best effects on the inhibition of neutrophils, the decrease rate of 59.9% and 57.4%, respectively. 5-aminosalicylic acid (5-ASA) is considered the drug of choice in conventional therapy for ulcerative colitis due to its safety and high efficacy [53]. However, the DX and HJ oils in this work showed better effects on decrease neutrophils in intestine of zebrafish compared with results treated by 5-ASA, as reported by [54]. And the concentrations of volatile oils were significantly lower than the substances used in some studies, such as c-phycocyanin and polysaccharides [32,54]. Furthermore, the effects on neutrophils in intestine of zebrafish treated by DX and HJ oils with different concentrations were also investigated. The oils of DX and HJ with high concentration (1 ng/mL) showed better inhibitory effect on neutrophils, suggesting that its anti-inflammatory effect on colitis is dose-dependent.

The effects of six volatile oil samples on the histopathological structure are shown in Figure 5E. Compared with the control group, a small number of epithelial cells in mucosa of intestine were necrotic, detached, and the nuclei were fragmented in the model group. However, after being treated with the oils of DX, GJ, HHJ, HJ, and RG, the intestine staining was uniform, the epithelial structure of mucosa was intact, the morphology and structure of epithelial cells were normal, regularly distributed. There were no significant abnormalities and inflammation of the lamina propria. In the intestine of zebrafish treated with XHX oils, it can be seen that some epithelial cells were degenerated, vacuolation occurred in the cytoplasm, and there were no significant abnormalities and inflammation of the lamina propria, as well. These results indicated that the volatile oils of six spices could improve the intestinal inflammation of zebrafish.

Terpenes are the main components of volatile oils contained in six spices and have the function of anti-inflammatory and anti-ulcer. The α-pinene, 1,8-cineol [55,56] contained in GJ oils, α-myrcene, benzaldehyde in RG oils [57], and anethole, estragole, limonene, α-pinene [58,59] in XHX oils showed excellent anti-inflammatory effects. Among them, α-pinene, α-myrcene, limonene, and sabinene are the common volatile compounds of six spices, which may be the active components of anti-inflammatory and anti-diarrhea.

Current methods to treat ulcerative colitis decrease inflammation by the use of anti-inflammatory agents and the reduction of oxidative stress [60]. Baker et al. [61] found that Lavender oil reduced intestinal tissue damage and decreased infiltration of neutrophils to the treatment of UC by reducing levels of TNF-α, IFN-γ, and IL-22. Among them, TNF-α plays a role in causing damage in the GI tract through its proinflammatory responses and can be regarded as targets for drug action. Angelica oil improves intestinal mucosal barrier damage in the inflammatory environment of mice with UC by inhibiting the expression of S100A8/A9 and the activation of its downstream TLR4/NF-κB signaling pathway [62]. Adakudugu, E.A. et al. [63] studied the protective effect of bergapten in acetic acid-induced colitis in rats. They found that bergapten enhances the clearance of neutrophils and macrophages from the site of inflammation and reduces oxidative stress by inhibition of reactive oxygen species (ROS). We can speculate from previous studies that the active components in volatile oils of the six spices may also treat ulcerative colitis through the abovementioned pathways, but the relevant mechanism needs to be further studied.

## 3. Materials and Methods

### 3.1. Materials

All materials for experiment were purchased from the market, and the detailed information is as follows: Black peppers were purchased from an online mall of Jing Dong, zanthoxylums were purchased from Anguo Guangsheng Trading Co., Ltd. (Anguo, China) while fennel, dried ginger, cinnamon, and cloves were purchased from Shandong Jianlian Decoction Pieces Co., Ltd. (Jinan, China). The samples were stored at ambient temperature before analysis by HS-GC-IMS. 3-Nitrobenzenesulfonic Acid (TNBS) was purchased from Wo En Biotechnology Co., Ltd. (Jinan, China).

The abbreviations of the six spices are as follows: HHJ (black peppers), HJ (zanthoxylums), XHX (fennel), GJ (dried ginger), RG (cinnamon), DX (cloves).

### 3.2. Methods

#### 3.2.1. HS-GC-IMS Analysis

Analysis of six samples was performed on Gas-phase Ion Mobility Spectrum Flavour Spec^®^ (Gesellschaft für Analytische Sensorsysteme mbH, Dortmund, Germany). The extraction and identification of VOCs in six spices were conducted according to the method that is normally used in the reported literature with some modification [43]. In brief, HHJ, GJ, RG, XHX, DX, and HJ were ground into powders, respectively. Then, 1.0 g of each sample was placed in 20 mL top-empty bottles, individually, and incubated at 60 °C for 15 min. Next, the temperature of the injection needle was set to 85 °C, and the injection volume was 100 μL. Then, an MXT-5 chromatography column (15 m × 0.53 mm × 1 µm) was used for gas chromatography separation at a column temperature of 60 °C. The temperature of IMS was set to 45 °C, and the carrier/drift gas was N_2_, with purity ≥ 99.999%. The flow rate of the carrier gas was set to 2 mL·min^−1^ (0–2 min), 2–100 mL·min^−1^ (2–20 min), 100 mL·min^−1^ (20–50 min), and the flow rate of drift gas was 150 mL·min^−1^. All analyses were performed in triplicate. The retention index (RI) of VOCs was calculated by using n-ketones C_4_–C_9_ as external references. VOCs were identified by comparing the RI and drift time (Dt) of the standard in the GC-IMS library.

#### 3.2.2. Extraction of Volatile Oils

An appropriate amount of powder of HHJ, GJ, RG, XHX, DX, and HJ was placed in six round-bottomed flasks individually and then extracted by refluxing using tenfold volumes of water for 8 h to obtain volatile oils.

#### 3.2.3. Animal Experiments

##### Animals Handling

Animal studies were approved by the Biology Institute, Qilu University of Technology of Animal Ethics Committee. Zebrafish Tg (*zlyz*: *EGFP*) were used in this study and kept in the lab of the Biology Institute, Shandong Academy of Sciences, according to the Westerfield [58] procedures. The adult zebrafish were divided into male and female groups before the day for egg retrieval and fed regularly with artificial granular bait and newly hatched artemia nauplii under a light/dark (14/10 h) cycle photoperiod. The male and female zebrafish were pooled together before light on the second day to obtain fertilized eggs. Then, the fertilized eggs were moved to zebrafish embryo water containing 5.0 mM of NaCl, 0.17 mM of KCl, and 0.16 mM of MgSO_4_, after being sterilized and washed, and cultured under controlled light at 28 °C.

##### TNBS-Induced Ulcerative Colitis in Zebrafish

A method similar to that previously reported [38] was adopted in this work to evaluate the effects of six volatile oils on mitigating colitis. In this experiment, the healthy zebrafish Tg (*zlyz*: *EGFP*) at 72 hpf were randomly distributed into several groups and placed in twenty-four-well plates, including a blank control group, TNBS model group, and volatile oil groups (GJ group: dried ginger oils-1 ng/mL; HHJ group: black pepper oils-1 ng/mL; XHX group: fennel oils-1 ng/mL; RG group: cinnamon oils-1 ng/mL; HJ groups: zanthoxylum oils-0.25, 0.5, and 1 ng/mL; DX groups: clove oils-0.25, 0.5, and 1 ng/mL). Each group had ten fish, with three repetitions. The blank control group zebrafish were incubated in fish water without TNBS, while the others were treated with fish water containing TNBS (60 μM) under the same conditions. After 72 h, the volatile oils of six spices were added to different groups in twenty-four-well plates, incubated for 24 h, and the zebrafish were anesthetized with tricaine (0.2%, *w/v*) for observation and image capture. An AXIO ZOOM.V16 stereo fluorescence microscope (CarlZeiss, Jena, Germany) and DP2-BSW image software (Olympus, SZX2-ILLTQ, Tokyo, Japan) were used to analyze the captured pictures to calculate the number of neutrophils at the intestinal of zebrafish.

##### Histopathological Observation of Zebrafish Intestinal

Ten zebrafish were randomly selected as per the method described in TNBS-Induced Ulcerative Colitis in Zebrafish Section, which have been treated with volatile oils (1 ng/mL) and fixed in 4% paraformaldehyde for 24 h. And then, the fixed zebrafish were dehydrated in gradient ethanol, immersed in xylene, and embedded in paraffin. Paraffin-embedded tissue sections were stained with hematoxylin and eosin (H.E.).

### 3.3. Statistical Analysis

Qualitative analysis of samples was performed using the NIST and IMS database built into the software. The GC-IMS fingerprints of each sample were obtained by the Gallery Plot plug-in of the Laboratory Analytical Viewer (LAV), which can compare the differences in VOCs of the samples intuitively and quantitatively. Dynamic Principal Component Analysis (Dynamic PCA) software (Version number: 2.2.1) was used to perform cluster analysis and similarity analysis on samples and to identify the unknown components. The Euclidean distance matrix among samples is calculated by “nearest neighbor” fingerprint analysis, which can retrieve the minimum distance to find the “nearest neighbor” and then observe relatively close group measurements compared to the more distant group. The experimental data were expressed as mean ± standard deviation (SD). GraphPad Prism 10.2 software (GraphPad Software Inc., San Diego, CA, USA) was used to analyze the data. Significant difference between the two groups was evaluated by Duncan′s test (*p* < 0.05).

## 4. Conclusions

In conclusion, the similarities and differences of volatile organic compounds in six warm spices can be analyzed by HS-GC-IMS quickly, non-destructively, and accurately, especially to the effective discrimination of isomers’ volatile compounds. And terpenes are major volatile components of spices which are related to the character flavor of them. Meanwhile, the volatile oils contained in six spices show excellent improvement of ulcerative colitis in zebrafish, and oils of clove and zanthoxylum have better effects under the same conditions. This study provides data support for identification and flavor analysis of warm spices based on volatile organic compounds. And the effects on ulcerative colitis shown by volatile oils in six spices are beneficial to the spices’ development as function food. But the mechanism of volatile oils in treating ulcerative colitis needs to be further studied in later work.

## Figures and Tables

**Table 1:** Results of the qualitative analysis of six spices.

No.	Compound	CAS	Formula	RI	Rt (s)	Dt (ms)	Odors	Assignment
1	β-caryophyllene	87-44-5	C_15_H_24_	1942.2	1705.813	1.43896	Wood, Spice	DX
2	Citral (M)	5392-40-5	C_10_H_16_O	1352.0	857.086	1.05297	Lemon	GJ
3	Citral (D)	5392-40-5	C_10_H_16_O	1348.0	851.372	1.62057		GJ
4	Decanal	112-31-2	C_10_H_20_O	1278.9	751.951	1.53787	Soap, Orange Peel, Tallow	HJ
5	Ethyl phenylacetate (M)	101-97-3	C_10_H_12_O_2_	1233.6	686.854	1.29494	Fruit, Sweet	HJ
6	Geraniol	106-24-1	C_10_H_18_O	1418.8	953.16	1.72739	Rose, Geranium	RG
7	α-terpineol	98-55-5	C_10_H_18_O	1294.5	774.439	1.22775	Oil, Anise, Mint	HJ
8	Methyl salicylate	119-36-8	C_8_H_8_O_3_	1236.5	691.092	1.20143	Peppermint	DX
9	Nerol	106-25-2	C_10_H_18_O	1250.1	710.566	1.22112	Sweet	XHX
10	Estragole	140-67-0	C_10_H_12_O	1249.8	710.126	1.2414	Licorice, Anise	XHX
11	Anethole	104-46-1	C_10_H_12_O	1453.6	1003.307	1.76643	Licorice, Anise	XHX
12	γ-octalactone	104-50-7	C_8_H_14_O_2_	1331.9	828.267	1.33601	Coconut	HJ
13	Ethyl phenylacetate (D)	101-97-3	C_10_H_12_O_2_	1227.7	678.42	1.78766	Fruit, Sweet	HJ
14	α-pinene oxide	1686-14-2	C_10_H_16_O	1084.3	472.131	1.22616	/	HHJ
15	Limonene	138-86-3	C_10_H_16_	1022.2	382.914	1.65342	Lemon, Orange	Common
16	α-phellandrene	99-83-2	C_10_H_16_	1006.3	360.049	1.68777	Turpentine, Mint, Spice	Common
17	*p*-mentha-1(7),8-diene	499-97-8	C_10_H_16_	999.5	350.251	1.72708	/	Common
18	α-myrcene	1686-30-2	C_10_H_16_	981.2	332.858	1.64744	/	Common
19	α-pinene	80-56-8	C_10_H_16_	933.5	292.194	1.67433	Pine, Turpentine	Common
20	β-ocimene	13877-91-3	C_10_H_16_	1047.0	418.595	1.71467	Citrus	HJ
21	δ-2-carene	4497-92-1	C_10_H_16_	992.1	342.167	1.72708	/	Common
22	Sabinene	3387-41-5	C_10_H_16_	970.3	323.55	1.64537	Pepper, Turpentine, Wood	Common
23	α-thujene	2867-05-2	C_10_H_16_	923.8	283.866	1.67433	Wood, Green, Herb	HJ
24	Heptaldehyde (M)	111-71-7	C_7_H_14_O	904.0	266.966	1.33343	Fat, Citrus, Rancid	HJ
25	Heptaldehyde (D)	111-71-7	C_7_H_14_O	904.2	267.167	1.69889		HJ
26	2-heptanone (M)	110-43-0	C_7_H_14_O	894.0	258.49	1.25987	Soap	DX
27	2-heptanone (D)	110-43-0	C_7_H_14_O	894.3	258.692	1.63798		DX
28	Trans-2-hexenal (M)	6728-26-3	C_6_H_10_O	850.7	234.679	1.18517	Apple, Green	HJ
29	Hexanal (M)	66-25-1	C_6_H_12_O	796.1	205.419	1.25413	Grass, Tallow, Fat	RG
30	Hexanal (D)	66-25-1	C_6_H_12_O	796.1	205.419	1.56903		RG
31	2-methylbutan-1-ol	137-32-6	C_5_H_12_O	739.3	180.832	1.22368	Wine, Onion	XHX
32	Pentanal (M)	110-62-3	C_5_H_10_O	702.3	165.858	1.18104	Almond, Malt, Pungent	GJ
33	2-methylbutyraldehyde (M)	96-17-3	C_5_H_10_O	673.4	156.18	1.15841	Cocoa, Almond	GJ
34	2-methylbutyraldehyde (D)	96-17-3	C_5_H_10_O	672.2	155.875	1.40207		GJ
35	3-methylbutyraldehyde (M)	590-86-3	C_5_H_10_O	660.9	152.819	1.16972	Malt	GJ
36	3-methylbutyraldehyde (D)	590-86-3	C_5_H_10_O	661.3	152.921	1.41338	Malt	GJ
37	Ethyl acetate (M)	141-78-6	C_4_H_8_O_2_	620.1	141.817	1.10098	Pineapple	DX
38	Ethyl acetate (D)	141-78-6	C_4_H_8_O_2_	616.7	140.901	1.34115		DX
39	2-methyl propanol (M)	78-83-1	C_4_H_10_O	634.5	145.688	1.16798	Wine, Solvent, Bitter	HHJ
40	Isobutyraldehyde (M)	78-84-2	C_4_H_8_O	570.1	128.328	1.10241	Pungent, Malt, Green	GJ
41	Isobutyraldehyde (D)	78-84-2	C_4_H_8_O	569.5	128.173	1.28738		GJ
42	Acetone	67-64-1	C_3_H_6_O	517.3	114.092	1.11918	Peppermint	Common
43	Ethanol	64-17-5	C_2_H_6_O	495.5	108.212	1.1268	Sweet	Common
44	2-propenylmethy disulfide (M)	2179-58-0	C_4_H_8_S_2_	913.8	275.404	1.10996	Garlic, Scallion	HJ
45	Propyl sulfide	111-47-7	C_6_H_14_S	886.8	254.025	1.15809	Garlic, Onion	DX
46	Isoamyl acetate (M)	123-92-2	C_7_H_14_O_2_	877.3	248.952	1.31112	Banana	HJ
47	Isoamyl acetate (D)	123-92-2	C_7_H_14_O_2_	877.3	248.952	1.75788		HJ
48	Furfural (M)	98-01-1	C_5_H_4_O_2_	831.1	224.171	1.08282	Bread, Almond, Sweet	DX
49	Furfural (D)	98-01-1	C_5_H_4_O_2_	830.6	223.909	1.3364		DX
50	Acetoin	513-86-0	C_4_H_8_O_2_	714.1	170.648	1.33487	Butter, Cream	HJ
51	Pentanal (D)	110-62-3	C_5_H_10_O	702.0	165.711	1.43156	Almond, Malt, Pungent	GJ
52	2-methyl propanol (D)	78-83-1	C_4_H_10_O	636.7	146.301	1.35994	Wine, Solvent, Bitter	HHJ
53	Methyl acetate	79-20-9	C_3_H_6_O_2_	543.6	121.18	1.2006	Sweet	DX
54	2-butanone (M)	78-93-3	C_4_H_8_O	604.8	137.693	1.06241	Ether	XHX
55	2-butanone (D)	78-93-3	C_4_H_8_O	603.7	137.391	1.25159		XHX
56	2-pentanone (M)	107-87-9	C_5_H_10_O	693.3	162.211	1.12262	Ether, Fruit	XHX
57	2-pentanone (D)	107-87-9	C_5_H_10_O	692.6	161.905	1.37694		XHX
58	Butyl sulfide (D)	544-40-1	C_8_H_18_S	1082.4	469.406	1.80755	Grass, Rose, Geranium	XHX
59	2-nonanone (D)	821-55-6	C_9_H_18_O	1096.8	490.16	1.88908	Hot milk, Soap, Green	DX
60	Benzaldehyde (M)	100-52-7	C_7_H_6_O	960.1	314.899	1.15391	Bitter Almond, Burnt Sugar, Cherry, Malt, Roasted Peppe	RG
61	2,6-dimethyl-4-heptanone	108-83-8	C_9_H_18_O	955.0	310.529	1.3238	Green	XHX
62	2-nonanone (M)	821-55-6	C_9_H_18_O	1094.3	486.528	1.4085	Hot milk, Soap, Green	DX
63	Butyl sulfide (M)	544-40-1	C_8_H_18_S	1083.5	471.075	1.29395	Grass, Rose, Geranium	XHX
64	Dehydrolinalool	29171-20-8	C_10_H_16_O	1064.0	442.936	1.72728	Sweet, Musk	HJ
65	Linalool	78-70-6	C_10_H_18_O	1105.5	502.654	1.75112	Flower, Lavender	HJ
66	1,8-cineol	470-82-6	C_10_H_18_O	1035.7	402.3	1.29295	Mint, Sweet	GJ
67	Benzaldehyde (D)	100-52-7	C_7_H_6_O	961.0	315.636	1.47649	Bitter Almond, Burnt Sugar, Cherry, Malt, Roasted Pepper	RG
68	Trans-β-ocimene	3779-61-1	C_10_H_16_	1037.9	405.41	1.22386	Sweet, Herb	Common
69	Styrene	100-42-5	C_8_H_8_	894.7	259.036	1.43117	Balsamic, Gasoline	RG
70	2,3-butanediol	513-85-9	C_4_H_10_O_2_	786.3	200.198	1.3698	Fruit, Onion	HJ
71	Methylpyrazine	109-08-0	C_5_H_6_N_2_	801.2	208.174	1.08018	Popcorn	GJ
72	2-propenylmethyl disulfide (D)	2179-58-0	C_4_H_8_S_2_	913.9	275.448	1.45652	Garlic, Scallion	HJ
73	Furfuryl alcohol	98-00-0	C_5_H_6_O_2_	857.2	238.183	1.1268	Burnt	HJ
74	Trans-2-hexenal (D)	6728-26-3	C_6_H_10_O	850.8	234.772	1.52517	Apple, Green	HJ
75	Isoamyl alcohol	123-51-3	C_5_H_12_O	736.0	179.515	1.49146	Whiskey, Malt, Burnt	HJ
76	1,4-dioxane	123-91-1	C_4_H_8_O_2_	681.2	158.295	1.32321	Ether	HJ
77	2,3-butanedione	431-03-8	C_4_H_6_O_2_	579.9	130.977	1.17453	Butter	HJ
78	Isopropyl alcohol	67-63-0	C_3_H_8_O	488.5	106.325	1.08636	Alcohol, Irritant	HJ
79	Limonene oxide	1195-92-2	C_10_H_16_O	1195.4	631.995	1.22306	Fruit	GJ
80	*p*-mentha-1,5-dien-8-ol	1686-20-0	C_10_H_16_O	1158.7	579.105	1.22254	/	GJ

Note: RI: retention index, Rt: retention time, Dt: drift time, the odor of compounds was obtained from http://www.flavornet.org/index.html (accessed on 7 August 2023) or https://www.chemicalbook.com/ProductIndex.aspx (accessed on 7 August 2023).

**Table 2:** Eigenvalue and variance contribution rate (%).

Principal Components	Eigenvalue	Variance Contribution Rate/%	Cumulative Variance Contribution Rate/%
1	47.722	34.241	34.241
2	28.393	23.228	57.469
3	20.630	15.954	73.423
4	15.068	10.383	83.806
5	12.188	8.470	92.276

**Table 3:** Factor score, principal component scores, and comprehensive scores of volatile components of black pepper and other samples.

Samples	Principal Component Scores					Comprehensive Scores
	Y1	Y2	Y3	Y4	Y5	
HHJ	−1.5509	2.0422	−3.8746	−1.4877	6.1415	−0.3510
DX	−6.2205	−8.9991	1.2182	2.5422	0.1818	−3.7001
RG	−2.3536	0.1096	−4.6950	−4.5146	−4.4557	−2.6483
GJ	−2.0804	3.3696	7.9618	−3.0126	0.1084	0.9056
HJ	13.6246	−2.6994	0.4132	0.3854	−0.1602	4.7252
XHX	−1.4191	6.1771	−1.0236	6.0874	−1.8157	−12.5029

**Table 4:** Statistical results of neutrophil numbers in each group ($\bar{x}\pm s$).

Group	Concentrations (ng/mL)	$\mathrm{Neutrophil}\;\mathrm{Numbers}\;(\bar{x}\pm s)$	Decrease Rate (%)
Control	-	5.7 ± 1.95	-
Model	-	31.2 ± 6.61 ^####^	-
DX oil	1	12.5 ± 3.81 ****	59.9
GJ oil	1	21.6 ± 6.83 ***	30.8
HHJ oil	1	15.4 ± 3.31 ****	50.6
HJ oil	1	13.3 ± 2.95 ****	57.4
RG oil	1	18.2 ± 2.57 ****	41.7
XHX oil	1	25.2 ± 8.69	19.2

Compared with control group: #### *p* < 0.0001, compared with model group: *** *p* < 0.001, **** *p* < 0.0001. “-” in the column of concentrations means there are no volatile oils in these group. “-” in the column of decrease rate means other group were compared with this group.

## Data Availability

The data that support the findings of this work are available from the corresponding author upon reasonable request.
